# Use of Electrical Household Appliances and Risk of All Types of Tumours: A Case-Control Study

**DOI:** 10.3390/medsci13020036

**Published:** 2025-04-01

**Authors:** Shabana Noori, Abdul Aleem, Imrana Niaz Sultan, Afrasiab Khan Tareen, Hayat Ullah, Muhammad Waseem Khan

**Affiliations:** 1 Department of Biotechnology, Faculty of Life Sciences and Informatics, Balochistan University of Information Technology Engineering and Management Sciences, Quetta 87300, Pakistan; 2 Department of Biosciences, COMSATS University, Islamabad 45550, Pakistan; 3 Biological Engineering, College of Engineering, Utah State University, Logan, UT 84322, USA

**Keywords:** extremely low frequency, electromagnetic field, household electrical appliances, powerlines, cancers

## Abstract

Introduction: The use of electrical appliances using extremely low frequency (ELF) electromagnetic fields (EMF) has increased in the past few years. These ELF MF are reported to be linked to several adverse health effects. However, only a couple of studies have been conducted on the association between risk of tumours and use of electronic devices using low frequency (LF) EMF. Methods: We studied the use of common household electrical appliances and suspected risk of tumours in a multi-hospital-based case-control study. In total, 316 patients were included in the final analysis. Results: The study results showed a below unity risk for most of the devices. A slight increased risk of tumour was observed for computer screen use OR: 1.13 (95% CI: 0.43–3.02) and use of microwave oven OR: 1.21 (95% CI: 0.36–4.04). We also had chance to investigate ELF MFs exposure association with tumour. Where we observed elevated odd ratios in individuals living near electricity transformer stations, with a statistically significant risk OR: 2.16 (95% CI: 1.30–3.59). However, the risk was below unity (OR: 0.98) in individuals residing close to powerlines. Conclusion: The current study serves as a pilot study of primary data and will be helpful in future epidemiological research studies on the topic in the region.

## 1. Introduction

Extremely low-frequency (ELF) electromagnetic fields (EMFs) are mainly associated with the production, transmission, and usage of electricity. Extremely low-frequency electromagnetic fields can cause a variety of alterations in the natural system of living organisms including human beings. Based on the findings of studies conducted on the topic, the International Agency for Research on Cancer (IARC) has classified ELF MFs as a possible human carcinogen [1].

Several significantly elevated risks are associated with exposure to ELF MF including adverse pregnancy outcomes [1,2], leukemia [3,4,5], acute lymphoblastic leukaemia [6], cancers of the central nervous system, solid tumours and brain tumours [4,5]. In addition, clinically recognized medical conditions such as cardiovascular disease breast cancers, kidney cancer and other types of cancer may also be associated with exposure to ELF MFs [7,8].

Also, apoptosis suppression [9], changes in cell-cycle distribution [10], alter gene and protein expression in the control of the cell cycle and the response to DNA damage [11] has also been reported. However, according to epidemiological studies, the connection between ELF MF and adverse health outcomes mainly tumours of different types have not been conclusively proven [6,12,13,14,15] and demands further investigation.

A few meta-analyses have reported a link between exposure to MF intensities of 0.1–2.36 µT and childhood leukaemia [16,17]. Comparing the highest exposure to lower exposures, B-lineage acute lymphoblastic leukaemia (B-ALL) risk was shown to be higher [18]. Records-based case-control studies of childhood leukaemia conducted across the state showed that there was a risk associated with higher exposure to MF produced near power lines [19,20].

On the other hand, electrical appliances in homes are one of the main sources of ELF EMFs exposure. Electrical appliances measurement studies have shown that the magnetic field strength is strongest near an appliance and rapidly declines with increasing distance from the appliance, and the intensity often depends on the type of transformer, motor, or heating element employed [21,22]. Magnetic fields from smaller, handheld equipment, such electric shavers and hair dryers, are typically higher than those from bigger appliances, like electric ovens [23].

The use of electronic devices using low frequency electromagnetic fields has increased in the past few years. However, to date only a couple of studies have been conducted on the association between risk of tumours and use of electronic devices using low frequency electromagnetic fields –demanding further studies [23,24]. These hospitals-based case control studies used questionnaires to examine whether appliances might be linked to adult brain tumours.

The multi-hospital based, case-control study presented here was conducted exploring the use of common household electrical appliances and suspected risk of various types of tumours. We evaluated the relationships between fourteen (14) frequently used household appliances and risk of tumours. Besides risk from electrical appliances, we also evaluated the risk of tumours from living close to mobile transmission stations, electricity power lines, and electricity transformer stations. Previous studies have reported the risk of tumours associated with living close to power lines [13] and transformer stations [6,12].

## 2. Materials and Methods

### 2.1. Study Subjects

A multi-center, hospital-based, case control study of all types of tumours was conducted at Center for Nuclear Medicine and Radiotherapy (CENAR), Quetta; Bolan Medical Complex (BMC), Quetta; Sandeman Provincial Hospital Quetta (SPHQ) and Sheikh Zayed hospital, Quetta. These hospitals (particularly CENAR), among others are serving as regional referral centers for the diagnosis and treatment of tumours in the geographic areas of Balochistan, Pakistan. Patients visiting the outpatient department (OPD) of these hospitals facilities were deemed eligible for the study according to the following criteria. Every patient visiting the OPD was included irrespective of the severity of the disease to avoid underrepresentation based on individual’s condition.

We included all types of tumours. The most reported types of tumours were esophageal (24 cases), breast (22 cases), brain (10 cases), ovary (9 cases), rectum (7 cases), leukemia (5 cases), osteosarcoma (5 cases), stomach (4 cases), cervical (4 cases), and lymphoma (4 cases).

The cases were all those individuals diagnosed with any type of tumour vising CENAR outpatient department (OPD) or OPD of oncology department of other included hospitals. The controls were selected from the general OPDs of SPHQ. Controls were individuals who had been visiting the OPD facility for the urology service; nephrology service; neurosurgery service; orthopedic service; obstetrics and gynecology service. Individuals who had been ever diagnosed with any type of cancer were not included in the study as controls. Also, individuals who have received radiotherapy and diagnostic radiation were not included in the study. In addition, maternity patients and individuals with diagnosis of Alzheimer’s disease, stroke, Parkinson’s disease or individuals relying on a cardiac pacemaker were excluded. Controls were matched to each case by gender and age (±5 years).

### 2.2. Data Collection

Interviews with the help of questionnaires, where each participant responded to face-to-face questionnaire administered by a trained interviewer were conducted in each participating hospital (at the time of a check-up) with both cases and controls. Beside selective household electric appliances use, the questionnaire included questions about their personal information, demographic information, socio-economic information, lifestyle, and past medical history. There were also questions about sunlight exposure, chemical exposure, welding fume exposure, smoking (both active and passive) and moist snuff, which is commonly used in the study region.

The interviews explored the participants’ use of selective household electrical appliances as a determining factor of their exposure to ELF MF. Each interview lasted about 30–45 min. The participants were questioned about the normal routine use and frequency of selective household electrical appliances including electric stove/heaters, electric shaver/razors (men), hand-held electric hair dryers, electric hair curlers (women), and humidifiers. These selective household electrical appliances are commonly used in the study population. It was also made sure to include only regular use of these electrical appliances in exposed group to confirm exposure variation and to avoid exposure misclassification. In addition to ELF EMF household appliances use, participants were asked about use of microwave ovens which operates at microwave frequency and the frequency range spans from ~300 MHz to 30 GHz (300 × 10^6^ Hz–30 × 10^9^ Hz) corresponding to a wavelength range of 1 m to 0.01 m (1000 mm–10 mm), respectively. There are also many other household appliances, however, the use of those appliances (e.g., alarm clocks) are not common in the study population and therefore not included in the study. We have tried to include every single household electrical appliance to avoid any chance of potential exposure misclassification by missing out on potentially relevant devices. The magnetic flux densities (in µT) at different distances associated with each studied household appliance is presented in Table 1.

The participants were also asked about the use and frequency of computer screens (monitors) and televisions, both in the workplace and at home, and about their videogame playing habits, both on monitors and on television sets.

As a proxy of ELF MFs exposure participants were also asked questions about their residential exposure to electricity transmission line and electricity transformer station. Participants were asked on the residence distance (in meters) to power lines of 400 kV. Participants residing within 300 m radius were considered as exposed and those living farther away were considered as controls.

### 2.3. Data Analysis

All the statistical analyses were performed using the IBM Statistical Package for the Social Science (SPSS) Version 25 (IBM Corp, Armonk NY, USA). Logistic regression was used to investigate the association between all types of tumours and selective electrical household appliances use. In the analysis, the adjustments were made to the full model for age, gender, smoking, nutritional status, residence, chemical, and pesticide exposure. Odds ratios (ORs) and corresponding 95% confidence intervals (CIs) were calculated. Statistical significance was determined based on *p* values < 0.05.

Characteristics of exposed and control groups were compared using Student’s *t*-test for continuous variables and Chi square test for binary response variables. Results were considered as statistically significant if *p* < 0.05.

## 3. Results

The study included a total of 316 participants (158 cases and 158 controls) including 142 (44.9%) men and 174 (55.1%) women. Both case and control groups had 71 men and 87 women. The mean age of the individuals at the time of data collection was 43.8 ± 19.67 years. The mean age of the cases was slightly higher 44.3 ± 19.05 than controls 43.2 ± 20.32; however not statistically significant. Also, the characteristics of case and control groups did not differ for gender, age, marital status, and moist snuff use. However, statistically significant differences were observed for residence, smoking, nutritional status, chemical exposure, and pesticide exposure between the groups. The characteristics of the study participants are shown in Table 2.

The Odd Ratio for all appliances combined (ever exposure) was below unity adjusted OR: 0.49 (95% CI: 0.31–0.77) and unadjusted OR: 0.74 (95% CI: 0.43–1.28), indicating that the risk of tumour was lower in the cases than controls. There was also no association between the use of electric shaver, electric stove and risk of tumours. An increased (not statistically significant) risk of tumour was observed between use of microwave oven and computer screen where the adjusted ORs were above unity OR: 1.21 (95% CI: 0.36–4.04) and OR: 1.13 (95% CI: 0.43–3.02) respectively (Table 3).

We also analyzed data for living close to electricity transmission line (power line) and transformer station. Elevated OR was observed for living close to transformer station where the risk was statistically significant OR: 2.16 (95% CI: 1.30–3.59). The risk of living close to powerlines was just below unity OR: 0.98 (95% CI: 0.63–1.52) (Table 4).

## 4. Discussion

The results of this study have suggested an increased risk of tumour from computer screen and microwave oven use. However, rather than a general increase in tumour risk, the data suggests decreased risk for other common household appliances including electric shaver, curling iron, electric stove or all electrical appliances together. The study findings further demonstrate that living close to transformer stations was associated with an increased risk of all types of tumours.

Self-reported use of electrical appliances and adult brain tumour risk has reported minimal proof that the use of a curling iron, heating pad, vibrating massager, electric blanket, heated waterbed, sound system, computer, television, humidifier, microwave or electric stove was associated with brain tumours [23]. The sex ratio of cases varied depending on the kind of tumour (male: female ratio = 1.2 for glioma, 0.3 for meningioma, and 0.6 for acoustic neuroma). There was a link between glioma and ever using a hair dryer OR: 1.7 (95% CI: 1.1–2.5), however, there was no indication that risk increased with usage. Meningioma was linked to electric shaver use in men OR: 10.9 (95% CI: 2.3–50), and ORs grew with cumulative usage minutes, even though the findings were based on just two non-exposed cases. The ORs for ever-using a microwave oven were non-significantly higher for all three types of brain tumour: glioma OR: 2.0 (95% CI: 0.9–4.8), meningioma OR: 1.5 (95% CI: 0.5–4.7), and acoustic neuroma OR: 1.9 (95% CI: 0.2–16).

With more years of electric shaver usage, the ORs for meningioma increased. The ORs did not consistently rise with cumulative use for hairdryers or microwave ovens. Long-term users of curling irons, electric heating pads, electric blankets, and sound systems with headsets were observed to have ORs lower than the unity. The ORs for glioma associated with regular use of electric blankets were not affected by the year of purchase or the temperature setting, and ORs remained below unity when the number of months of usage of electric blankets increased annually [23].

Case-control research study on primary malignant brain cancer in adults looked at the risk posed by common home appliances. Regular usage of any of these electrical devices was found to have no risk of brain cancers [24]. The study found no significant link between home appliance use and brain cancer occurrence, even in high-frequency use situations. For instance, 11% of cases used a computer monitor more than 5 h per day, while 15.5% of controls used the same frequency (OR = 0.7). Those who used home computer monitors reported similar results, out of 328 cases, 18 (5.5%) reported using them more than 8 h a week, while 23 (28.1%) of the 284 controls reported using them the same amount of the time (OR = 0.7). Three out of 328 cases (0.9%) and seven out of 284 controls (2.5%) reported using electric space heaters for longer than four hours per day (OR = 0.4). 54 of the 284 controls (18.0%) reported using an electric blanket on a regular basis for at least a year, compared to 64 of the 326 responding cases (19.6%) who reported using one (OR = 1.1). A similar pattern was seen in the usage of handheld electric hair dryers, 130 of 284 controls (45.8%) and 153 of 328 cases (46.7%) reported using one on a regular basis for at least a year (OR = 1.0). Sex-specific stratification did not suggest any probability of increased risk until males reported owning an electric-dial bedside clock for a minimum of a year. Results showed no statistically significant difference in the reporting of such exposure between 68 of 192 male cases (35.4%) and 43 of 157 male controls (27.4%) (OR = 1.5, *p*-value > 0.10) [24].

Studies have also examined the use of electrical appliances, such as heated water beds and electric blankets, in relation to the development of brain cancers. The usage of a heated waterbed OR: 0.8 (95% CI: 0.3–1.9) or an electric blanket OR: 0.5 (95% CI: 0.2–1.4) by the child was not linked to a higher risk of developing a brain tumour [27]. In prenatal exposure to heated water beds OR: 0.9 (95% Cl: 0.6–1.3) or electric blankets OR: 0.9 (95% CI: 0.6–1.2) did not increase the chance of developing a brain tumour. Children whose mothers reported using throughout their pregnancies did not have an increased risk of developing brain cancer. Similar findings were found for exposure to children, showing no correlation between brain cancer and usage of electric blankets OR: 1.0 (95% CI: 0.6–1.7) or heated water beds OR: 1.2 (95% CI: 0.7–2.0) [28].

Household electrical appliances are one of the main sources of ELF EMFs exposure. According to results from measurement studies, the magnetic field strength is strongest near an appliance and rapidly declines with increasing distance from the appliance. Many of these common household electrical appliances, i.e., hair dryer and electric razor are used at very close distance (at the distance of 3 cm) where the magnetic field flux densities are very high. According to measurement studies, the magnetic flux density for hair dryer at the distance of 3 cm is 6–2000 µT and that for the electric razor is 15–1500 µT, at the distance of 30 cm. Similarly, the normal operating distance for microwave oven, electric cooker and computer screen is 30 cm where the magnetic flux densities are 4–8 µT, 0.15–0.5 µT and <0.01 µT, respectively.

According to our study results, tumour was found to be more widespread in rural participants as compared to urban participants. Since rural population has less exposure to electrical appliances, other factors may be the cause of tumour such as use of chemical fertilizers, insecticides, or pesticides and nutritional status and many others. A recent systematic analysis has reported that individuals from rural settings have worse prognoses and quality of life due to travel distance, hindering optimal cancer screening and treatment [29]. Nutritional status significantly impacts survival, treatment completion, and healthcare consumption, with interventions only partially able to mitigate these negative outcomes. Pesticides, widely used in agriculture and domestic settings, are a significant cause of disorders in humans and wildlife due to their mode of exposure and concentration [30].

Besides smoking, use of moist snuff is common in the studied population. It is also observed that people smoke in public places, indoors and during commuting in the public transport [31]. Among various types of smokeless tobacco, moist snuff is most popular among young people and appears to have the most sophisticated engineering for regulating the dosage of nicotine, making the product itself the main factor in determining how much nicotine is consumed when it is placed between the cheek and the gum. The bloodstream received a significant amount of nicotine quickly. The pH of the snuff product in aqueous suspension affected both how much nicotine was absorbed and how quickly. Snuff use for a long period of time can cause a variety of harmful health impacts, including cardiovascular disease, gingivitis, and mouth cancer [32].

Concerning ELF MFs exposure (from powerlines and transformer stations), our study results are in line with previous studies conducted in different settings. A cohort research study on adult brain tumours and hematological malignancies among occupants of structures using indoor transformer stations reported risk of acute lymphocytic leukemia (ALL) associated with exposure to ELF MFs. However, this result for ALL was based on only four exposed cases. Also, the study reported increased risk for glioma. The risk for hematological neoplasms and meningioma decreased rather than an increase [6]. Similarly for skin cancers, the study reported that the overall risks of melanoma or squamous cell carcinoma were not found to be affected by MF exposure. However, early childhood exposure was reported to be associated with increased risk of melanoma [12].

These both studies were based on the ELF MFs exposure from indoor transformer stations, common in European countries and in Finland. The exposure data was used from a unique database of ELF MF exposure; Database of Finnish Buildings with Indoor Transformer Stations (DaFBITS). A disadvantage from this approach was that there was no information about exposure to ELF MFs sources other than transformer stations. However, other residential sources are not likely to be important, as the transformer stations are dominating sources in these kinds of apartments [33].

Despite its strengths, the current study has several limitations. Firstly, the current study has relatively small sample size. Due to the low number of tumour cases and low number of participants with appliances use, the analysis could not reveal significant results. Secondly, being a case-control study, self-reported data might introduce reporting bias due to social desirability. Thirdly, exposure is assessed via self-report, which might result in a potential recall bias that could lead to differential exposure misclassification between cases and controls. However, we have only selected common household appliances and the chances of recall (if any) would be minimal. Potential confounders (not included in this study e.g., socioeconomic status etc.) and some potentially important mediators (e.g., stress coping and offline social support) should be considered in future studies. Finally, the study is not based on a specific type of tumour (we have included all types of tumours). For example, a couple of studies have been conducted on adult brain tumour and home electrical appliances exposure to ELF MFs [23,24]. Results indicate, brain tumour risk is not likely to be increased by extremely low frequency electromagnetic fields from routinely used home electrical appliances. Although, currently, there is no known mechanism by which these low frequency EMFs could damage DNA and cause cancer. However, even a small increase in risk would be of clinical importance given how widespread exposure to these fields is. Concerning living close to powerlines and transformer stations, spot or 24/48 h measurements in the residencies of the participants would have resulted in a more objectively measured exposure approximation.

In this study, there is no hair dryer use in control, only 5 individuals used hair dryer in tumour cases. We do not have strong evidence of hair dryers causing tumour due to a very small number of cases. However, further study is needed to explore the effects of EMF from hair dryers on human health. Also, in this study, no curling iron was used in controls whereas only 2 individuals in cases used it; these could be due to poverty however, the socioeconomic position of the controls varied in this study (not reported here).

The strength of our research study is that this case-control study is conducted in a resource poor area of the country. The study includes people who do not have access to different electronic devices such as TV, computer screens (some even have not even seen computer screens). Also, there are individuals in the remote areas with access to electricity facility. These are the unique settings (where exposure misclassification may not affect the results), where we can hardly find these kinds of people worldwide, particularly in developed countries.

Also, it is a strength of the present study that all diagnosed cases were reviewed centrally. The participation rate was high among the eligible cases (98%) and controls (100%) which helps in controlling potential selection bias.

## 5. Conclusions

The study overall does not support an association between household electrical appliances use and risk of tumour. The finding of an excess risk of tumour for computer screen use and use of microwave oven is intriguing and demands further studies. Furthermore, the study observed elevated risk in individuals living near electricity transformer stations but not powerlines. The study also concludes that due to the low number of study participants, these findings might be affected by chance bias.

## Figures and Tables

**Table 1:** Magnetic field strengths (in µT) of common household electrical appliances at the distance of 3 cm, 30 cm and 1 m [22,25,26].

Electrical Appliance	Magnetic Flux Density (µT) at 3 cm Distance	Magnetic Flux Density (µT) at 30 cm Distance	Magnetic Flux Density (µT) at 1 m Distance
Electric shaver	15–1500	0.08–9	0.01–0.3
Microwave oven	73–200	4–8	0.25–0.6
Electric stove	1–50	0.15–0.5	0.01–0.04
Computer screen	0.5–30	<0.01	<0.01
Television	2.5–50	0.04–2	0.01–0.15

**Table 2:** Characteristics of study participants (N = 316), divided into two groups: tumour cases and control respondents.

		Tumour N	%	Control N	%	p *
Age, Mean (SD)		44.3 (19.05)		43.2 (20.32)		0.605
Gender						
	Male	142	44.9	142	44.9	
	Female	174	55.1	174	55.1	1.000
Marital status						
	No	35	22.2	30	19.0	
	Yes	123	77.8	128	81.0	0.487
Residence						
	Urban	49	31.0	90	56.9	
	Rural	109	69.0	68	43.1	<0.001
Smoking						
	Never	143	90.5	153	96.8	
	Ever	15	9.5	5	3.2	0.021
Moist snuff						
	Never	132	83.5	119	75.3	
	Ever	26	16.5	39	24.7	0.070
Nutritional status						
	Poor	51	32.3	8	5.1	
	Medium	91	57.6	149	94.3	
	Good	16	10.1	1	0.6	<0.001
Chemical exposure						
	Never	151	95.6	157	99.4	
	Ever	7	4.4	1	0.6	0.032
Pesticide exposure						
	Never	132	83.5	152	96.2	
	Ever	26	16.5	6	3.8	<0.001

* *p* value for continuous variables is based on Student’s *t*-test and *p* value for categorical variables are based on Chi square test.

**Table 3:** Odd ratios (OR) and 95% confidence intervals (95% CIs) for risk of cancer among number of cases and the number of controls who have ever used different types of electrical appliances.

Appliance Use				Adjusted Analysis *		
		Tumour Cases N	Controls N	OR	95% Confidence Interval	*p*
Electrical appliance exposure (any)						
	Never	92	64			
	Ever	66	94	0.49	0.31–0.77	0.002
Electric shaver (men)						
	Never	157	156			
	Ever	1	2	0.50	0.05–5.54	0.570
Microwave oven						
	Never	152	153			
	Ever	6	5	1.21	0.36–4.04	0.759
Electric stove						
	Never	135	108			
	Ever	23	50	0.37	0.21–0.64	0.002
Computer screen						
	Never	149	150			
	Ever	9	8	1.13	0.43–3.02	0.803
Television						
	Never	102	93			
	Ever	56	65	0.79	0.50–1.24	0.298

* Adjusted for: Age, gender, smoking, nutritional status, residence, chemical and pesticide exposure.

**Table 4:** Odd ratios (OR) and 95% confidence intervals (95% CIs) for living close to mobile transmission station, powerlines, transformer station and risk of cancer.

				Adjusted Analysis *		
		Tumour Cases N	Controls N	OR	95% Confidence Interval	*p*
Electric transmission line						
	<300 m	69	68			
	≥300 m	89	90	0.98	0.63–1.52	0.910
Transformer station						
	<300 m	102	126			
	≥300 m	56	32	2.16	1.30–3.59	0.003

* Adjusted for: Age, gender, smoking, nutritional status, residence, chemical and pesticide exposure.

## Data Availability

All data generated or analyzed during this study are included in this published article.
